# EHMTI-0148. Perivascular spaces and headache: a population-based imaging study (MRI HUNT)

**DOI:** 10.1186/1129-2377-15-S1-F12

**Published:** 2014-09-18

**Authors:** AK Husøy, LM Honningsvåg, AK Håberg, K Hagen, M Linde, M Gårseth, LJ Stovner

**Affiliations:** 1 Neuroscience, Norwegian University of Science and Technology, Trondheim, Norway; 2 Radiology, Levanger Hospital, Trondheim, Norway

## Background

In three former studies headache sufferers (in particular migraineurs) had more perivascular spaces (PVS) on MRI than headache-free.

## Aim

To evaluate the association between headache and PVS in a relatively large and population-based imaging study with a blinded design.

## Method

The study was part of a large longitudinal epidemiological study (Nord-Trøndelag Health Survey (HUNT)). The 1006 participants were 50-65 years at inclusion, had participated in all previous HUNT studies (1, 2 and 3), and had been randomly selected to a population-based imaging study of the head (MRI-HUNT, 2007-2009). The number of dilated PVS in the basal ganglia (BG) and hemispheric white matter (HWM) was compared in headache sufferers (migraine with and without aura, non-migrainous headache) and headache-free. Both cross-sectional and longitudinal analyses were performed.

## Results

The cross-sectional analysis showed that migraineurs without aura in HUNT 3 had fewer PVS than headache-free (OR=0.84, 95% CI=0.75-0.95, P-value=0.003) in BG. In the longitudinal analysis those with migraine in only HUNT 2 were found to have fewer PVS than headache-free (OR=0.98, 95% CI=0.96-1.00, P-value=0.049) in HWM. There was no relation between PVS and any other headache types.

## Conclusion

In contrast to the findings of previous studies the present study showed no increase in number of dilated PVS among headache sufferers. Fewer PVS in migraineurs without aura may be an incidental finding.

No conflict of interest.

